# Cost-consequence analysis of tocilizumab versus adalimumab and etanercept among rheumatoid arthritis patients in Saudi Arabia: a single-center study

**DOI:** 10.1186/s12962-024-00522-7

**Published:** 2024-02-14

**Authors:** Areej S. Albahdal, Amjad M. Alotaibi, Miteb A. Alanazi, Norah Abanmy, Monira Alwhaibi, Yazed AlRuthia

**Affiliations:** 1 Department of Clinical Pharmacy, College of Pharmacy, King Saud University, 11451 Riyadh, P.O. Box 2454, Saudi Arabia; 2 Department of Pharmacy, King Khalid University Hospital, 12372 Riyadh, P.O. Box 3145, Saudi Arabia; 3 Pharmacoeconomics Research Unit, Department of Clinical Pharmacy, College of Pharmacy, King Saud University, 11451 Riyadh, P.O. Box 2454, Saudi Arabia

**Keywords:** Tocilizumab, Etanercept, Adalimumab, Arthritis, Rheumatoid, Cost analysis

## Abstract

**Background:**

The study aimed to examine the direct medical cost and impact of tocilizumab (TOZ) versus adalimumab (ADM) and etanercept (ETC) on reducing the levels of two inflammatory markers (e.g., C-reactive protein (CRP) and erythrocyte sedimentation rate (ESR)) among patients with rheumatoid arthritis (RA) using real-world data from Saudi Arabia.

**Method:**

This was a single-center retrospective cohort study in which data for biologic-naïve RA patients aged ≥ 18 years and treated with TOZ, ADM, ETC were retrieved from the electronic medical records (EMRs) of a university-affiliated tertiary care center in Riyadh, Saudi Arabia. Patients were followed up at least one year after the treatment initiation. Bottom-up microcosting was utilized to estimate the direct medical costs. Additionally, inverse probability treatment weighting and bootstrapping with 10,000 replications were conducted to generate 95% confidence levels for costs and the mean reductions in CRP and ESR levels.

**Results:**

The number of patients who met the inclusion criteria and were included in the analysis was 150 patients (TOZ (*n* = 56), ADM (*n* = 41), ETC (*n* = 53)). Patients on TOZ had 3.96 mg/L (95% CI: -0.229–4.95) and 11.21 mm/hr (95% CI: 10.28–18.11) higher mean reductions in the CRP and ESR levels compared to their counterparts on ADM, ETC, respectively. However, this was associated with mean annual incremental costs of USD 10,087.88 (95% CI: 9494.50–11,441.63) in all cost-effectiveness bootstrap distributions.

**Conclusion:**

Tocilizumab has shown better effectiveness in reducing the levels of CRP and ESR but with higher costs. Future studies should examine whether the reduction of these two inflammatory markers is associated with quality-adjusted life years (QALYs) gains.

## Introduction

Rheumatoid arthritis (RA) is an inflammatory, autoimmune disease that attacks the immune system, causing inflammation and persistent pain, swelling, stiffness, functional impairment, and psychological distress [1]. The global point-prevalence of RA is estimated to be 0.45% (95% CI: 0.38–0.53%) with an annual incidence rate of 0.02–0.05% [2, 3]. However, the incidence rates vary considerably in different parts of the world, with the highest pooled prevalence of 0.69% (95% CI: 0.47–0.95%) based on a recently published systematic review and meta-analysis [3]. In Saudi Arabia, there are no accurate statistics about the prevalence rate of RA. However, some single-centered studies with small sample sizes that examined the prevalence of RA in some geographic regions in Saudi Arabia found a relatively higher prevalence of RA, with a reported prevalence of 2.2 per 1000 people [4, 5]. In addition, females are two to three times more likely to be affected with RA than their male counterparts [6]. However, the human leukocyte antigen (HLA) locus continues to be the most significant genetic risk factor associated with RA [7]. Other identified risk factors were found to be associated with a higher risk of RA, such as family history of RA, cigarette smoking, exposure to silica and textile dust, periodontitis, nutritional difficulties, obesity, hormonal imbalance, and low educational attainment [8, 9].

The goal of RA treatment is to achieve remission, defined as a Disease Activity Score in 28 joints (DAS28) of less than 2.6 or low disease activity (e.g., DAS28 < 3.2) if remission is not possible [10]. Moreover, preventing joint damage, disability, and other systemic manifestations, including cardiovascular damage, are essential treatment goals [1, 11]. Currently used medications in the treatment of RA include non-steroidal anti-inflammatory drugs (NSAIDs), glucocorticoids, and conventional and targeted or biological disease-modifying anti-rheumatic drugs (DMARDs) [12]. However, methotrexate remains the most commonly used medication for managing RA, especially in the early stages, due to its low cost and well-established effectiveness and safety [13]. On the other hand, biologic DMARDs such as tumor necrosis factor inhibitors (TNFi), costimulatory inhibitors, interleukin-6 inhibitors, and B-cell depleting drugs are effective in treating RA. However, due to their high cost, they are only recommended for patients who have failed to respond to or are intolerant to conventional DMARDs, such as methotrexate [12]. Therefore, biologic DMARDs have been continuously evaluated for cost-effectiveness [12, 14]. One of the biologic DMARDs that demonstrated its effectiveness in the management of active and progressive RA is tocilizumab (TOZ), which is an interleukin-6 (IL-6) inhibitor with a low immunogenicity risk, flexible route of administration (intravenous (IV) and subcutaneous (SC)), and can be self-administered subcutaneously once-weekly [13]. Over the last decade, extensive several clinical trials and observational studies have firmly established the short and long-term efficacy and safety of IV and SC TOZ as monotherapy or combination therapy in adults with moderate to severe RA, including both early-stage and established RA, with both formulations (IV and SC) exhibiting similar efficacy [15–19]. Despite the previous treatment, TOZ resulted in a fast and persistent improvement in RA signs and symptoms [17, 18, 20]. In addition, TOZ achieved a rapid and long-lasting remission among more patients than TNFi and abatacept and had a favorable safety profile [20, 21]. Moreover, the use of TOZ as a first-line biologic monotherapy for patients with active RA who failed to respond to one or more DMARDs and were intolerant to methotrexate was deemed cost-effective from the public healthcare payer’s perspective in Greece [22]. In another study aimed to reduce the uncertainty about the use of biologic DMARDs for managing moderate to severe RA in Finland, the use of TOZ in combination with methotrexate resulted in higher incremental quality-adjusted life years (QALYs) gain. It was deemed cost-effective in comparison to adalimumab (ADM) + methotrexate or etanercept (ETC) + methotrexate [23]. In another cost-utility analysis of TOZ versus other biologic DMARDs (ETC, ADM, or infliximab) among patients with inadequate response to traditional DMARDs that was conducted from the payer’s perspective in Italy, the replacement of anti-TNF-α treatments with TOZ resulted in cost saving over the patient’s lifetime and realized more QALYs compared to the standard of care [24].

In Saudi Arabia, the management of RA is costly mainly due to the high acquisition cost of biologic DMARDs [5]. According to a single-center study that estimated the direct medical cost (medications, lab and diagnostics, hospitalization, procedures, visits, emergency, and physical therapy) of RA in Saudi Arabia, the average annual cost per patient was estimated to be USD 10,292.26 ± 814.66 [25]. Moreover, it is essential to consider other indirect costs related to absenteeism (time off from work), presenteeism (work performance influenced by health issues), work disability, and early retirement [13]. Therefore, examining the cost-effectiveness of biologic DMARDs is instrumental in informing the decision makers about the value for money of these expensive therapies. Although the cost-effectiveness of TOZ has been examined against different biologics and combination therapies using patient-level simulation models [14, 24, 26], very few studies examined its cost-effectiveness using real-world data [20, 27]. Unfortunately, no study has evaluated TOZ against other biologics for managing RA in Saudi Arabia due to several barriers, such as lack of access to valid clinical data and lack of a national cost-effectiveness threshold [28]. Therefore, examining the cost-effectiveness of TOZ versus other commonly used biologics, such as ADM and ETC, for the management of RA is of crucial importance to healthcare policymakers in Saudi Arabia at a time of comprehensive healthcare reform [29].

## Methods

### Study design and population

The study was a retrospective, single-centered study. Data on adult patients (≥ 18 yrs.) with RA who were treated with TOZ, ADM, and ETC for ≥ 12 months were retrieved from EMRs of a university-affiliated tertiary care center in Riyadh, Saudi Arabia. Patients with cancer, any incidence of serious infections post-biological treatment initiation, and pregnant or breastfeeding women during the follow-up were excluded. Furthermore, patients on methotrexate or other traditional DMARDs (sulfasalazine, hydroxychloroquine, and leflunomide) were excluded. Patients who were not biologic-naïve (treated before or being treated with either TOZ, ADM, or ETC before the follow-up) were excluded. The cost-consequence analysis was conducted from the perspective of public healthcare payers in Saudi Arabia, in which only direct medical costs (e.g., lab tests, imaging studies, clinic visits, medications, etc.…) were accounted for in the analysis. The analysis did not include indirect costs, such as missed days from school or work.

### Data collection and study variables

In order to examine the effectiveness of biologic DMARDs, inflammatory markers (C-reactive protein (CRP) and erythrocyte sedimentation rate (ESR)) were used due to the absence of documented valid effectiveness outcomes, such as DAS28 [30]. These biomarkers were studied and linked to RA disease progression [31]. Therefore, they assessed the RA disease progression by measuring the mean reductions in the CRP and ESR between baseline (i.e., before the initiation of the treatment with TOZ, ETC, or ADM) and follow-up periods. Two pharmacy interns reviewed the medical charts of patients who met the inclusion criteria and collected all the relevant variables. Patients were followed retrospectively using the EMRs from treatment initiation with TOZ, ADM, ETC and 12 months later. Patient’s demographics (age, gender), weight, treatment, and disease duration, baseline and follow-up CRP and ESR levels, other prescription drugs, such as glucocorticoids (e.g., prednisone), and non-steroidal anti-inflammatory drugs (NSAIDS), comorbidities (hypertension, diabetes mellitus, dyslipidemia, cardiovascular diseases, asthma, hypo/hyperthyroidism, depression, obstructive apnea), pain and morning stiffness were collected as well. Micro-costing was used to capture all utilized health services, including lab tests, imaging studies, hospitalization, emergency department visits, outpatient clinic visits, and nursing and physician fees throughout follow-up. Data on the cost of different health services were retrieved from the Saudi Ministry of Health cost center.

### Descriptive statistics and multiple linear regressions

The number of RA patients treated with DMARDs in the study setting was 501 patients. However, the minimum sample size needed was estimated to be 133 patients based on an effect size of Cohen’s f^2^ = 0.06 (the proportion of variance explained by the linear model relative to unexplained variance), α = 0.05, β = 0.2, power of 80%, and up to 9 predictor variables for multiple linear regression. The baseline characteristics of the patients were presented using means, standard deviations, frequencies, and percentages. One-way ANOVA, Chi-square, Fisher’s exact tests were conducted, as appropriate, to compare the baseline characteristics of the patients on TOZ versus their counterparts on ADM, ETC. Multiple linear regressions were conducted to examine the impact of TOZ versus ADM, ETC on CRP and ESR levels 12 months after treatment, controlling for age, gender, treatment duration, disease duration, baseline CRP, baseline ESR, number of comorbidities, and glucocorticoids.

### Cost-consequence and sensitivity analysis

The effectiveness of TOZ against ADM, ETC was compared using the mean reductions in both CRP and ESR levels. On the other hand, the mean annual treatment cost of TOZ versus ADM, ETC were compared. All costs were expressed in United States Dollars (USD). In addition, propensity score-based inverse probability of treatment weights based on patients’ characteristics, such as age, gender, treatment duration, disease duration, use of glucocorticoids, baseline CRP and ESR levels, and number of comorbidities, was conducted to minimize the confounding effect. To examine the uncertainty about cost and effectiveness difference between TOZ versus ADM, ETC, non-parametric bootstrapping with 10,000 replications was conducted to generate the 95% confidence intervals (e.g., 95% CI). All statistical analyses were performed using SAS® version 9.4 (SAS® Institute, Cary, NC, USA).

## Results

### Patient characteristics

**Fig. 1:**
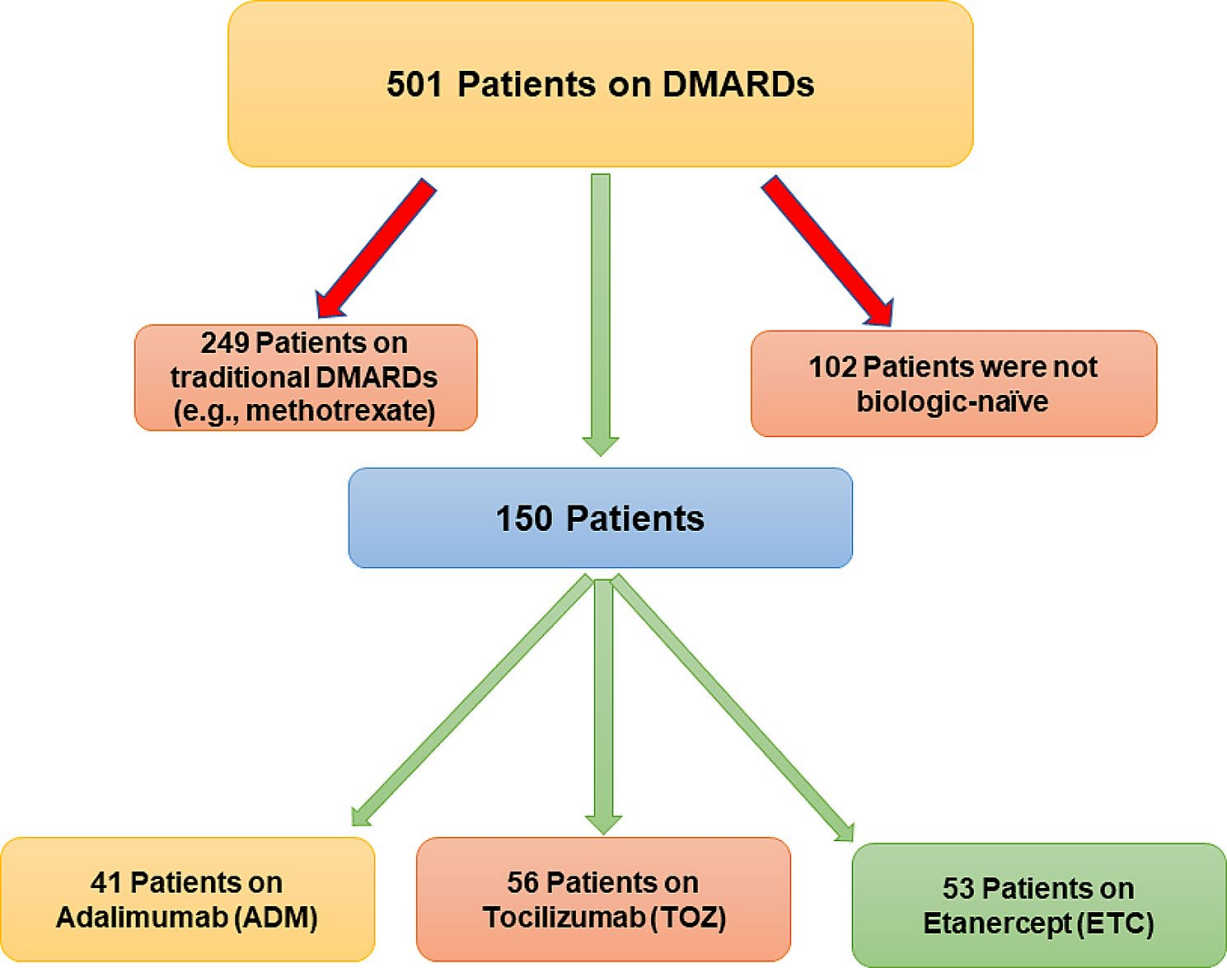
Patient recruitment scheme

Although more than 500 EMRs for patients with RA have been reviewed, only 150 patients met the inclusion criteria and were included in the analysis (Fig. 1). Of those, 56 patients were on TOZ, 41 on ADM, and 53 on ETC. Patients’ mean age was 53 years; most of them were females (93.33%). Even though patients on TOZ, ADM, ETC mean duration of illness were not significantly different (11.35 yrs.), the duration of treatment for patients on TOZ (2.42 yrs.) was significantly (*p*-value < 0.0001) shorter than their counterparts on ADM (4.42 yrs.), ETC (6.01 yrs.) as shown in Table 1. Only 10% of patients received glucocorticoids (e.g., prednisone), and almost 20% were treated with NSAIDs (e.g., celecoxib), with no significant difference between patients treated on TOZ, ADM, ETC. The most commonly encountered chronic health conditions were diabetes (26.66%), hypertension (30.67%), dyslipidemia (16%), hypothyroidism (21.33%), and asthma (9.33%), with no significant difference between patients treated with TOZ, ADM, ETC. However, obstructive apnea, morning stiffness, and pain were more commonly encountered among patients on ADM, ETC in comparison to their counterparts on TOZ. Although the mean baseline ESR level was lower among patients on TOZ (37.39 mm/hr) compared to their counterparts on ADM (44.97 mm/hr), ETC (49.41 mm/hr) (*p*-value = 0.0004), their mean baseline CRP level (7.55 mg/dL) was not significantly different. Other baseline lab values were not different between patients on TOZ, ADM, ETC, as shown in Table 1.

**Table 1:** Patient baseline characteristics (*n* = 150)

Characteristic	ADM (*n* = 41)	ETC (*n* = 53)	TOZ (*n* = 56)	*p*-value	Total
Gender, N (%)					
Female	39 (95.12)	46 (86.79)	55 (98.21)	0.0546	140(93.33)
Male	2 (4.88)	7 (13.21)	1 (1.79)		10(6.67)
Age (yrs.), mean ± SD	53.73 ± 16.04	54.58 ± 13.03	53.14 ± 10.37	0.847	53.81 ± 12.98
Weight (kg), mean + SD	75.60 ± 17.24	72.70 ± 17.91	77.84 ± 14.63	0.2721	75.41 ± 16.60
Duration of illness (yrs.), mean ± SD	10.07 ± 6.16	12.79 ± 6.50	10.91 ± 5.62	0.1032	11.35 ± 6.16
Duration of treatment (yrs.), mean ± SD	4.42 ± 2.55	6.01 ± 2.48	2.42 ± 1.52	< 0.0001	4.24 ± 2.67
Glucocorticoids, N (%)	5 (12.20)	4 (7.55)	6 (10.71)	0.7877	15(10.00)
NSAIDS, N (%)	10 (24.39)	10 (18.87)	9 (16.07)	0.5702	29(19.33)
Comorbidities, N (%)					
Diabetes	12 (29.27)	15 (28.30)	13 (23.21)	0.7982	40(26.66)
Hypertension	16 (39.02)	16 (30.19)	14 (25)	0.3277	46(30.67)
Cardiovascular disease (CVD)	3 (7.32)	2 (3.77)	0	0.0764	5(3.33)
Dyslipidemia	5 (12.20)	8 (15.09)	11 (19.64)	0.6138	24(16)
Asthma	4 (9.76)	5 (9.43)	5 (8.93)	1.0000	14(9.33)
Hypothyroidism	10 (24.39)	13 (24.53)	9 (16.07)	0.487	32(21.33)
Hyperthyroidism	0 (0)	0 (0)	1 (1.79)	1.000	1(0.67)
Depression	1 (2.44)	0	0	0.2733	1(0.67)
Obstructive apnea	4 (9.76)	0	0	0.005	4(2.67)
Morning stiffness, (N%)	11 (26.83)	8 (15.09)	6 (10.71)	0.1102	25(16.67)
Pain, (N%)	11 (26.83)	16 (30.19)	11 (19.64)	0.4407	38(25.33)
Labs, mean ± SD					
ESR (mm/hr), mean ± SD	44.97 ± 23.67	49.41 ± 29.57	37.39 ± 27.20	0.0004	43.71 ± 27.49
CRP (mg/dL), mean ± SD	8.75 ± 9.006	6.76 ± 9.84	7.41 ± 9.80	0.1947	7.55 ± 9.57
ALT (U/L)	26.93 ± 15.08	27.05 ± 52.91	38.82 ± 52.91	0.1357	31.42 ± 35.04
AST (U/L)	17.95 ± 9.09	19.88 ± 11.46	22.43 ± 14.02	0.1837	20.31 ± 11.99
HCT (%)	37.24 ± 3.92	39.03 ± 3.51	40.24 ± 11.33	0.1566	38.99 ± 7.56
HGB (g/L)	121.53 ± 14.3	126.56 ± 20.33	126.41 ± 22.47	0.3950	125.13 ± 19.76
Pts (platelets X 10^9^/L)	293.65 ± 102.59	276.16 ± 75.64	260.12 ± 93.45	0.1977	274.96 ± 90.69
RBC (X10^12^/L)	4.51 ± 0.49	4.67 ± 0.51	4.57 ± 0.43	0.2287	4.59 ± 0.48
WBC (X10^9^/L)	7.28 ± 2.72	6.52 ± 1.69	6.20 ± 2.53	0.0768	6.61 ± 2.35
Cholesterol (mmol/L)	4.75 ± 0.82	4.81 ± 1.03	5.28 ± 1.12	0.0855	4.99 ± 1.03
Triglyceride (mmol/L)	1.28 ± 0.58	1.28 ± 0.4	1.38 ± 0.65	0.5513	1.32 ± 0.56
HDL (mmol/L)	1.43 ± 0.26	1.39 ± 0.21	1.44 ± 0.26	0.5692	1.43 ± 0.25
LDL (mmol/L)	2.83 ± 0.51	2.98 ± 0.60	3.22 ± 1.31	0.1136	3.04 ± 0.92

*p < 0.05

### Regression models for the association between TOZ and the mean reductions in ESR and CRP levels

**Fig. 2:**
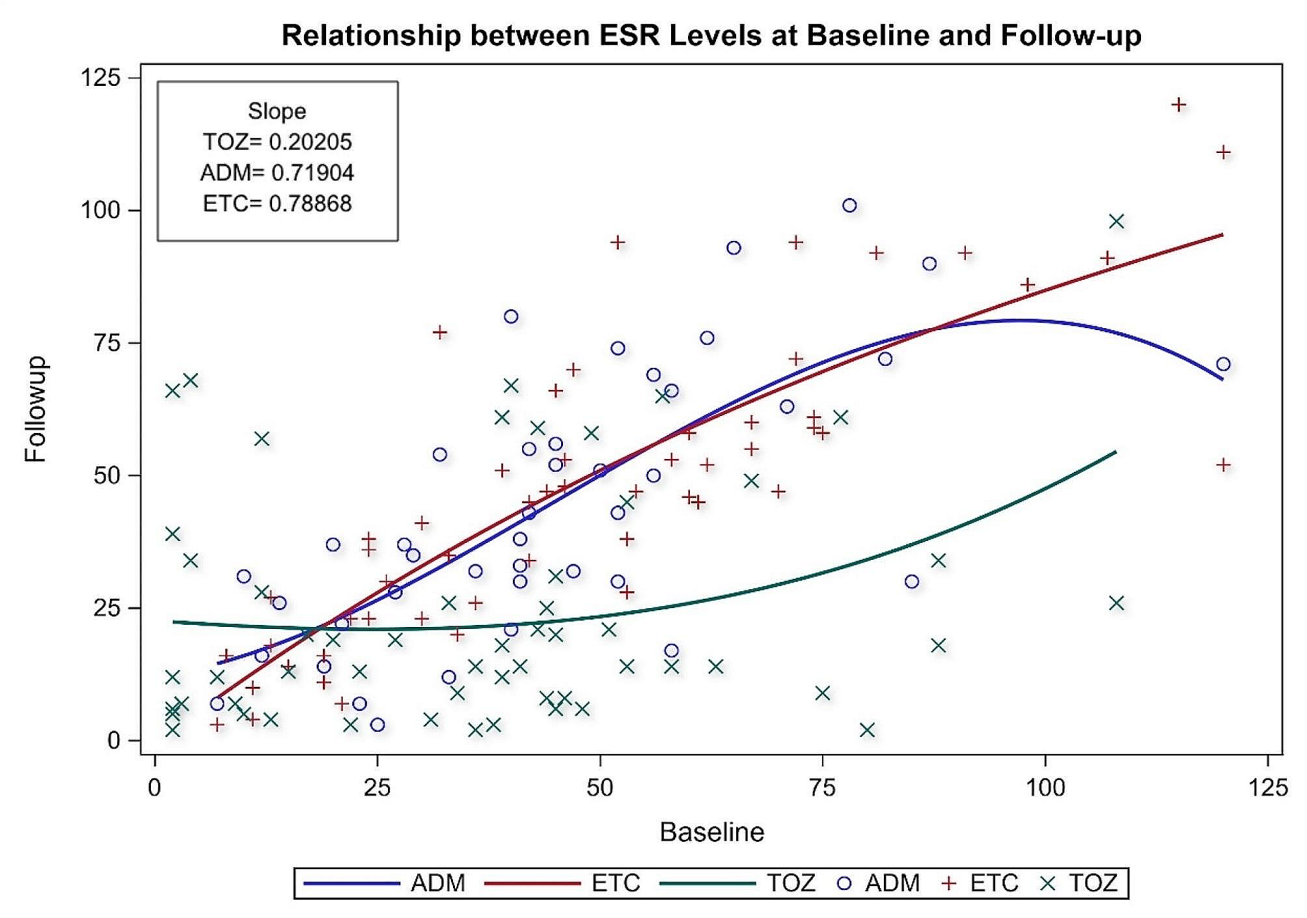
Regression line for the relationship between ESR levels at baseline and follow-up

**Fig. 3:**
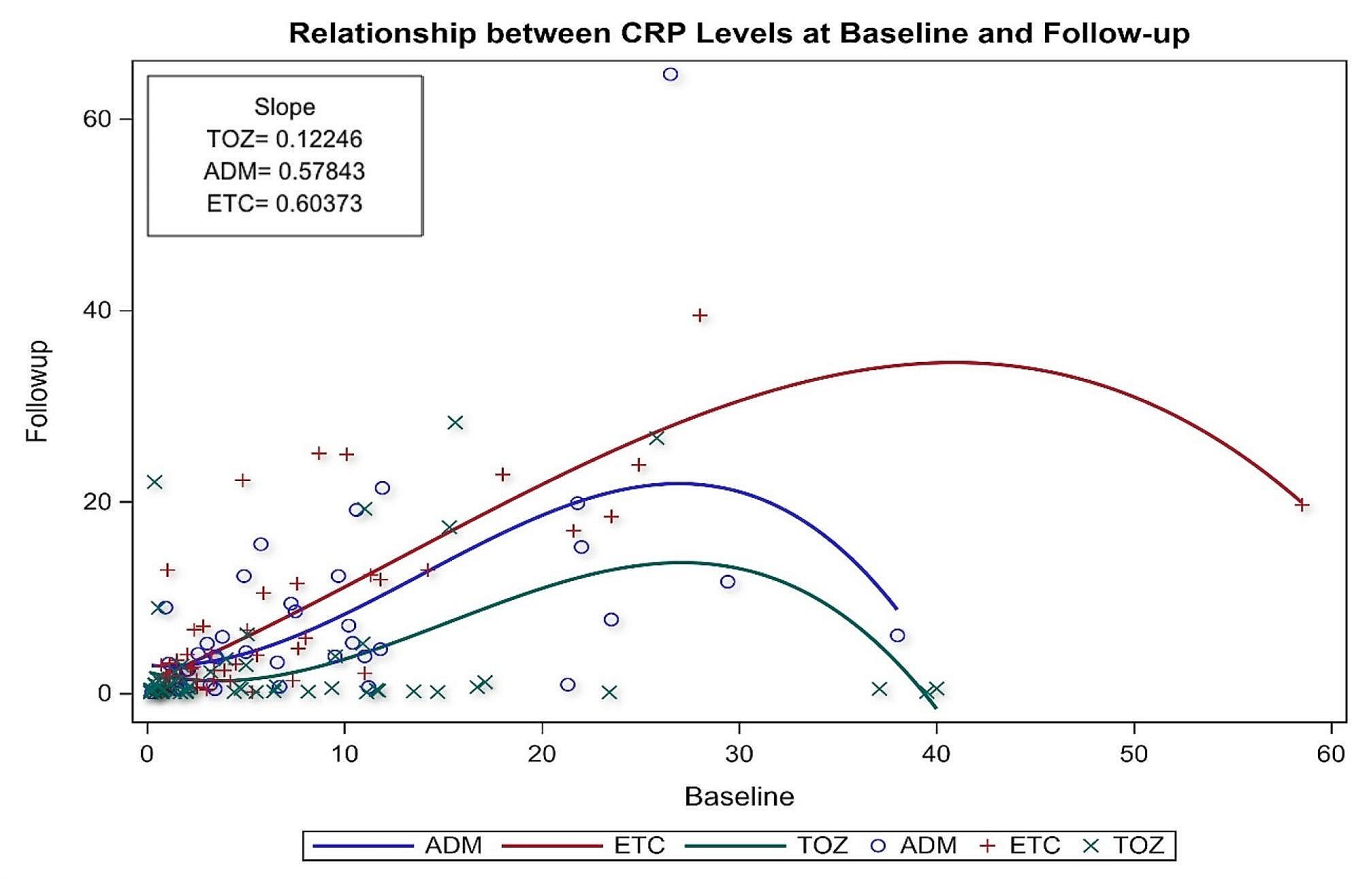
Regression line for the relationship between CRP levels at baseline and follow-up

Each one-unit increase in the baseline ESR level is associated with 0.202, 0.719, and 0.788 unit increases in the follow-up ESR levels for patients on TOZ, ADM, and ETC, as illustrated in Fig. 2. For CRP level, each one unit increase in the baseline CRP level is associated with 0.122, 0.57, and 0.60 unit increase in the follow-up CRP levels for patients on TOZ, ADM, ETC, as illustrated in Fig. 3, which shows that patients on TOZ are more likely to have more significant reductions in their ESR and CRP levels on follow-up than their counterparts on ADM, ETC. In addition, patients treated with TOZ for ≥ 12 months were more likely than their counterparts (ADM, ETC) to have more significant reductions in their ESR levels (β = 11.32, 95% CI=[3.22**–**19.41], *p*-value = 0.0065) controlling for age, gender, treatment and disease durations, baseline ESR and CRP levels, number of comorbidities, and use of glucocorticoids. Moreover, patients with higher baseline ESR levels were more likely to have more significant reductions in their ESR levels on the follow-up (β = 0.44, 95% CI= [0.309**–**0.573], *p*-value < 0.0001). On the other hand, patients with long treatment durations were less likely to have reductions in their ESR levels compared to those with short treatment durations (β=-1.77, 95% CI= [3.33 **-**– 0.21], *p*-value = 0.0261). Likewise, patients treated with glucocorticoids were less likely to have reductions in their ESR levels than their counterparts who were not treated with glucocorticoids (β=-15.57, 95% CI= [–26.68 **-**– 4.46], *p*-value = 0.0064) as shown in Table 2. Concerning the impact on CRP level, patients on TOZ were more likely to have more significant reductions in their CRP levels compared to their counterparts on ADM, ETC (β = 3.83, 95% CI= [1.12–6.55], *p*-value = 0.006) controlling for age, gender, treatment and disease durations, baseline CRP and ESR levels, number of comorbidities, and use of glucocorticoids as shown in Table 3. In addition, patients with higher baseline CRP were more likely to have more significant reductions in their follow-up CRP levels (β = 0.73, 95% CI= [0.600–0.852], *p*-value < 0.0001). On the other hand, patients with high baseline ESR were less likely to have reductions in their CRP levels compared to their counterparts with low baseline ESR (β=-0.069, 95% CI= [–0.114 **-**– 0.025], *p*-value = 0.0022). Moreover, the use of glucocorticoids was associated with lower reductions in CRP levels (β=-0.069, 95% CI= [–0.114 **-**– 0.025], *p*-value = 0.0022).

**Table 2:** Multiple linear regression for the impact of TOZ on the mean difference in erythrocyte sedimentation rate (ESR) level (ESR _baseline_-ESR _follow−up_)

variable	Parameter Estimates	*p*-value	95% Confidence Limits	
TOZ vs. ADM or ETC	11.31884	0.0065*	3.22486	19.41283
Age	-0.14195	0.3154	-0.42052	0.13661
Female vs. male	9.47349	0.1624	-3.86311	22.81009
Treatment Duration	-1.77115	0.0261*	-3.32801	-0.21429
Disease Duration	0.27866	0.3319	-0.28717	0.84450
Baseline CRP	0.19126	0.3165	-0.18489	0.56741
Baseline ESR	0.44103	< 0.0001*	0.30911	0.57296
number of comorbidities	0.49072	0.3807	-0.61261	1.59405
glucocorticoids	-15.57322	0.0064*	-26.68695	-4.45948

*p < 0.05

**Table 3:** Multiple linear regression for the impact of TOZ on the mean difference in C-reactive protein (CRP) level (CRP _baseline_-CRP _follow−up_)

variable	Parameter Estimates	*p*-value	95% Confidence Limits	
TOZ vs. ADM or ETC	3.83094	0.0060*	1.11550	6.54638
Age	-0.01850	0.6961	-0.11195	0.07495
Female vs. male	-2.94758	0.1949	-7.42186	1.52670
Treatment Duration	0.21941	0.4077	-0.30290	0.74172
Disease Duration	0.00758	0.9372	-0.18225	0.19741
baseline CRP	0.72634	< 0.0001*	0.60015	0.85254
baseline ESR	-0.06988	0.0022*	-0.11414	-0.02562
number of comorbidities	0.24384	0.1949	-0.12631	0.61400
glucocorticoids	-11.42014	< 0.0001*	-15.14867	-7.69160

*p < 0.05

### Cost-consequence analysis of TOZ versus ADM and ETC

**Fig. 4:**
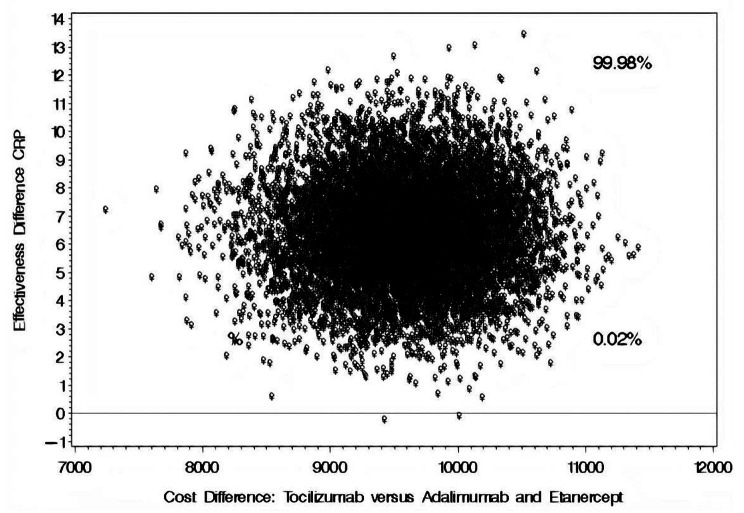
Bootstrap distribution of cost effectiveness of tocilizumab versus ADM and ETC for CRP level reduction

**Fig. 5:**
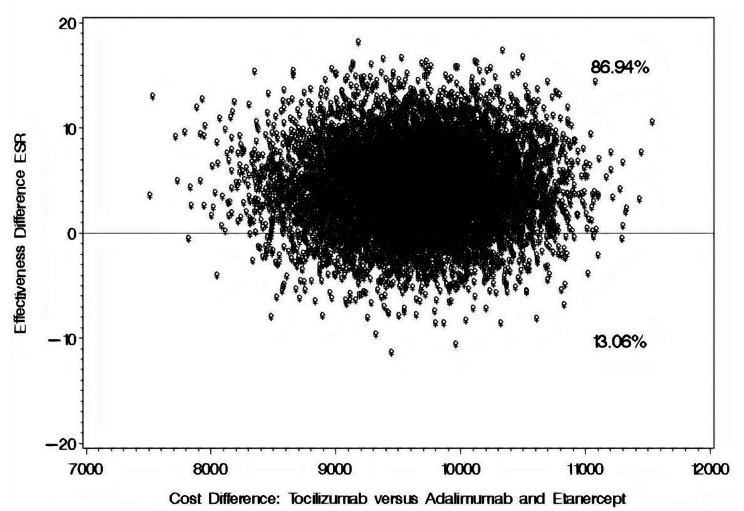
Bootstrap distribution of cost effectiveness of TOZ versus ADM or ETC for ESR level reduction

The mean difference in annual direct medical cost for patients on TOZ versus their counterparts on ADM or ETC was USD 10,087.88 [95% CI: 9,494.50–11,441.63], as shown in Table 4. The mean reductions in CRP for patients on TOZ and their counterparts on ADM or ETC were 4.34 mg/dL and 0.38 mg/dL, respectively, resulting in a mean difference of 3.96 mg/dL [95% CI: − 0.23–4.96] in favor of TOZ. According to the cost-effectiveness bootstrap distributions, the use of TOZ versus ADM, ETC for the management of RA will result in a more significant reduction in the CRP levels but at a higher cost in 99.98% of the bootstrap distributions, as depicted in Fig. 4. On the other hand, the use of TOZ versus ADM or ETC resulted, on average, in an incremental reduction of 11.21 mm/hr [95% CI: 10.28–18.11] in patients’ ESR levels in 86.94% of the bootstrap distributions but at a higher cost, as depicted in Fig. 5.

**Table 4:** The mean effectiveness rates and annual costs of TOZ versus ADM and ETC

	TOZ	ADM and ETC	Mean difference (95% Confidence interval)
Mean cost of treatment (USD)	16,945.26 ± 3,113.04	6,857.38 ± 3,113.04	10,087.88 (9,494.50**–**11,441.63)
Mean effectiveness rate of CRP	4.34 ± 10.75	0.38 ± 8.79	3.96 (1.611–4.96)
Mean effectiveness rate of ESR	12.73 ± 30.88	1.52 ± 18.09	11.2108 (10.28**–**18.11 )

## Discussion

The use of TOZ for managing RA has shown to be effective in controlling disease progression and improving physical function and quality of life [16, 18–20]. In this study, TOZ has shown a better inhibitory effect on CRP and ESR, two inflammatory biomarkers associated with RA progression and joint damage, compared to ADM, ETC [31, 32]. The more significant reductions in both CRP and ESR among patients on TOZ compared to ADM, ETC remained significant even after controlling for multiple covariates, such as age, gender, baseline CRP and ESR levels, disease and treatment durations, number of comorbidities, and use of glucocorticoids (e.g., prednisone), which indicates the superior efficacy of TOZ versus tumor necrosis factor-α inhibitors (TNFi), such as ADM, ETC. This is consistent with other previously published studies that used real-world data and compared TOZ to TNFi [16, 18, 20]. However, this comes at a higher cost as the mean annual treatment cost of TOZ is almost 2.5 times higher than TNFi (ADM, ETC) (USD 16,945.26 *versus* USD 6,857.38). If translated into QALY gains, these findings align with multiple cost-effectiveness studies conducted from the perspective of public healthcare payers and applied simulation models to project lifetime costs and outcomes among different hypothetical cohorts using inputs from clinical trials [14, 22, 23, 26, 27]. Therefore, the high incremental cost of TOZ versus TNFi that was found in this study can be justified if the use of TOZ against TNFi (ADM, ETC) is associated with QALY gains and is below the most recently published national cost-effectiveness threshold, which states that health technology is cost-effective if the incremental cost effectiveness ratio does not exceed USD 13,333 per each QALY gained [33].

Other interesting findings were also observed. The reductions in the ESR levels among RA patients seem to diminish with time as TOZ exhibits its maximal inhibitory effect on inflammatory biomarkers, such as ESR, in the first few weeks of treatment [34]. Moreover, glucocorticoids, such as prednisone and dexamethasone, were associated with lower reductions in CRP and ESR levels, which is interesting since many studies have demonstrated a substantial reduction in the CRP and ESR levels among patients with RA without necessarily impacting disease progression [35, 36]. However, in a recently published programmatic controlled clinical trial that examined the impact of low-dose prednisolone on disease activity and different inflammatory biomarkers among elderly patients (> 65 yrs.) with RA, the study found a beneficial effect of low-dose prednisolone as an add-on therapy on disease activity and joint damage progression. However, this was at the expense of an increased risk of non-severe infections and adverse events [37]. These findings refute the negative impact of glucocorticoids on the inflammatory biomarkers, such as CRP and ESR, that were found in this study. Nevertheless, the characteristics of the patients in the clinical trial differed from those in this study, whereby only 14% of the patients in the clinical trials were treated with biologics, and all of them were elderly [37].

Although this is the first study to the best of our knowledge that economically evaluated TOZ versus other commonly used TNFi (ADM, ETC) for managing RA using real-world data in Saudi Arabia, it has multiple limitations that must be acknowledged, first, the study used surrogate laboratory markers (CRP and ESR) instead of more comprehensive and valid measures, such as DAS28 [30], to assess the effectiveness of TOZ versus ADM, ETC. Although elevated inflammatory markers, such as CRP, have been associated with disease progression and joint damage, they are not disease-specific [31]. Secondly, this was a single-center study, which limits the generalizability of the results. Thirdly, data were retrieved from EMRs, and information bias cannot be ruled out. Additionally, the study did not examine the impact of TOZ and ADM or ETC on HRQoL to examine whether the use of TOZ would result in QALY gains, which was mainly due to the lack of validated utility estimates for different patient populations in Saudi Arabia [28]. Finally, no price sensitivity analysis was conducted to check whether the change in the acquisition cost of TOZ, ADM, ETC would change the cost-effectiveness of TOZ since the analysis was conducted based on real-world data and from the perspective of public health payers that procure their requested quantities of TOZ, ADM, ETC from a single public procurer with a fixed-price tender.

## Conclusions

Tocilizumab has shown better effectiveness in reducing the levels of CRP and ESR with reasonable incremental cost compared to TNFi (ADM, ETC) among a sample of RA patients in Saudi Arabia. Although elevated CRP and ESR levels have been associated with disease progression and joint damage among RA patients, some studies still question their validity as valid surrogate markers for RA disease progression. Therefore, future studies with larger sample sizes and more robust designs should be conducted to validate the findings of this study and examine whether higher CRP and ESR levels are associated with poor HRQoL.
